# Ascending aorta dilatation common feature which correlate with left ventricular hypertrophy in fabry disease

**DOI:** 10.1186/1532-429X-13-S1-P307

**Published:** 2011-02-02

**Authors:** Mourad Bensalah, Ferreira Antonio, Cedric Collin, Pierre Boutouyrie, Dominique Germain, Elie Mousseaux

**Affiliations:** 1 Hospital Georges Pompidou, Paris, France; 2 Hospital Raymond Poincare Garches, Paris, France

## Purpose

To describe the prevalence and characteristics of aortic root dilatation in male patients with Fabry disease (FD) and to assess its relationships with left ventricular remodelling.

## Methods and results

Fourty-four adult males with FD (age: 38.1±11.3 years) and 44 healthy male controls matched for age were included. The diameters of the ascending and descending aorta were measured by magnetic resonance imaging (MRI) at the level of the sinuses of Valsalva, sinotubular jonction, tubular portion, aortic arch and descending aorta. Cardiac geometry and properties were also assessed by MRI.

Dilatation of the ascending aorta was found in 40.9% of the patients with FD and was predominantly located at the sinuses of Valsalva (38.2±4.6 vs. 32.4±3.1 mm, *P*<0.0001). The dilatation was associated with increased left ventricular mass (LVM), independently of age and presence of hypertension. In multivariate analysis, LVM was the main determinant of the sinus diameter (R^2^=13.6%, *P*<0.05). When assessing the regional remodelling of the ascending aorta by a mean diameter estimated at three different levels, both LVM (R^2^=30.4%, *P*=0.0001) and renal failure (R^2^=7.3%, *P*=0.0297) were independently associated with aortic dilatation.

## Conclusion

Dilatation of the ascending aorta is a common finding in male patients with FD, witch correlate with left ventricular hypertrophy. This further expands the phenotype expression of Fabry disease.

